# T‐Cell Prolymphocytic Leukaemia With Isolated Pulmonary Manifestation

**DOI:** 10.1002/rcr2.70238

**Published:** 2025-06-10

**Authors:** Yu Kobayashi, Hiromi Tomioka, Rina Sakai, Hideaki Goto

**Affiliations:** ^1^ Department of Respiratory Medicine Kobe City Medical Center West Hospital Kobe Japan; ^2^ Department of Hematology and Oncology Kobe University Hospital Kobe Japan; ^3^ Department of Hematology and Oncology Hyogo Prefectural Harima‐Himeji General Medical Center Himeji Japan

**Keywords:** case report, ground‐glass opacities, isolated pulmonary manifestation, pulmonary involvement, T‐cell prolymphocytic leukaemia

## Abstract

T‐cell prolymphocytic leukaemia (T‐PLL) is a rare haematologic malignancy. We report a case of T‐PLL with isolated pulmonary manifestation, confirmed through video‐assisted thoracoscopic surgery biopsy. This case highlights that isolated lung infiltration in T‐PLL may reflect disease progression, requiring careful evaluation and timely therapeutic intervention.

## Graphical abstract

A 69‐year‐old male was diagnosed with T‐cell prolymphocytic leukaemia (T‐PLL) with isolated pulmonary involvement, confirmed through thoracoscopic lung biopsy. This rare presentation emphasises the importance of considering T‐PLL in patients with unexplained pulmonary infiltrates and highlights the need for timely pathological confirmation.

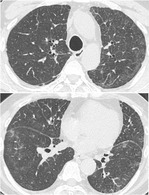

A 69‐year‐old male presented with a persistent cough lasting over 1 year. His medical history included hypertension. Computed tomography revealed diffuse ground‐glass opacities and interlobular septal thickening in both lungs (Figure 1). There was no evidence of hepatosplenomegaly or lymphadenopathy. Laboratory findings showed leucocytosis (15.29 × 10^9^/L) with 37.5% atypical lymphocytes. C‐reactive protein levels were negative; however, soluble interleukin‐2 (IL‐2) levels were elevated to 4200 U/mL. Human T‐cell leukaemia virus‐1 antibody test results were negative. Bone marrow examination revealed similar atypical lymphocytes to those found in peripheral blood, with immunophenotyping results for CD2, CD3, CD5, CD7 and CD52. T‐cell receptor β‐chain Cβ1 rearrangement was detected. Immunohistochemistry of the bone marrow tissue was positive for CD3, CD4 and CD5 but negative for CD8 and terminal deoxynucleotidyl transferase (Figure 2). These findings confirmed T‐cell prolymphocytic leukaemia (T‐PLL) [1]. Following diagnosis, thoracoscopic lung biopsy revealed infiltration of identical cells, with pulmonary involvement confirmed by immunophenotyping, immunohistochemistry (Figure 3), and detection of T‐cell receptor β‐chain Cβ1 rearrangement (Figure 4).

**FIGURE 1:**
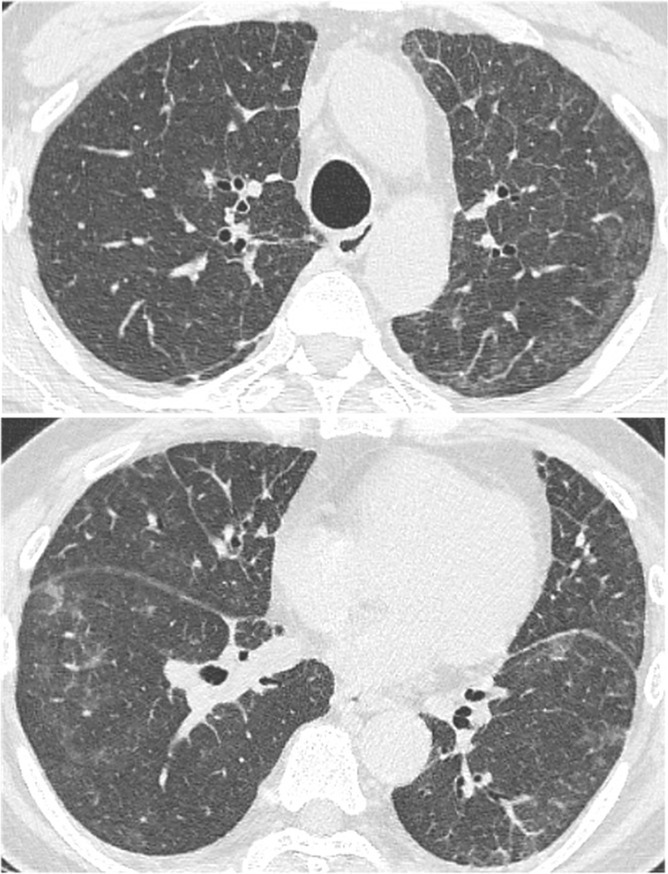
High‐resolution computed tomography of both lungs showing diffuse ground‐glass opacities and thickened interlobular septa in the peripheral regions of both lungs.

**FIGURE 2:**
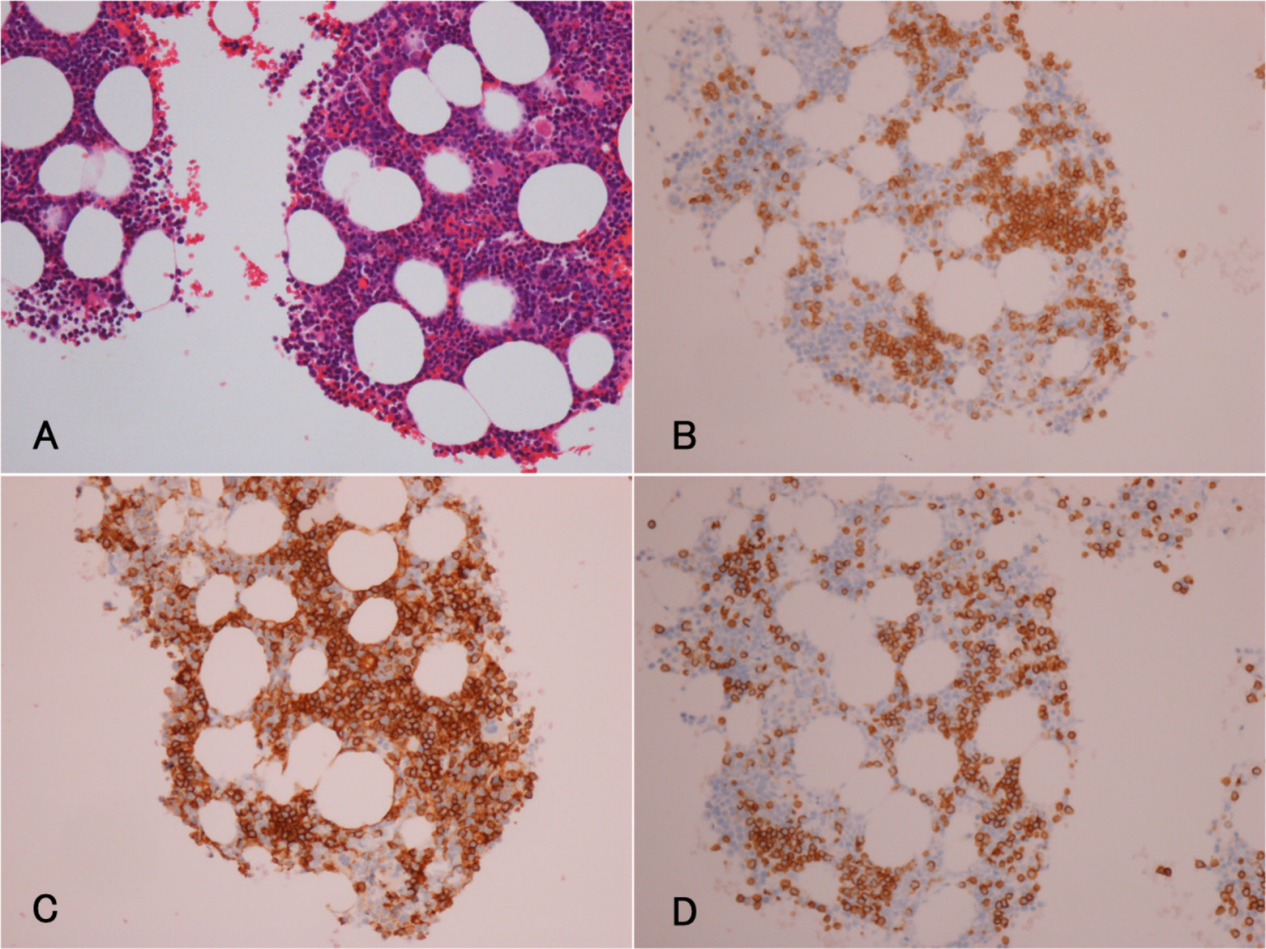
(A) Haematoxylin‐and‐eosin staining of bone marrow showing diffuse infiltrative proliferation of atypical lymphocytes. Immunostaining revealed: (B) CD3‐positive T cells; (C) numerous CD4‐positive T cells; and (D) CD5‐positive T cells.

**FIGURE 3:**
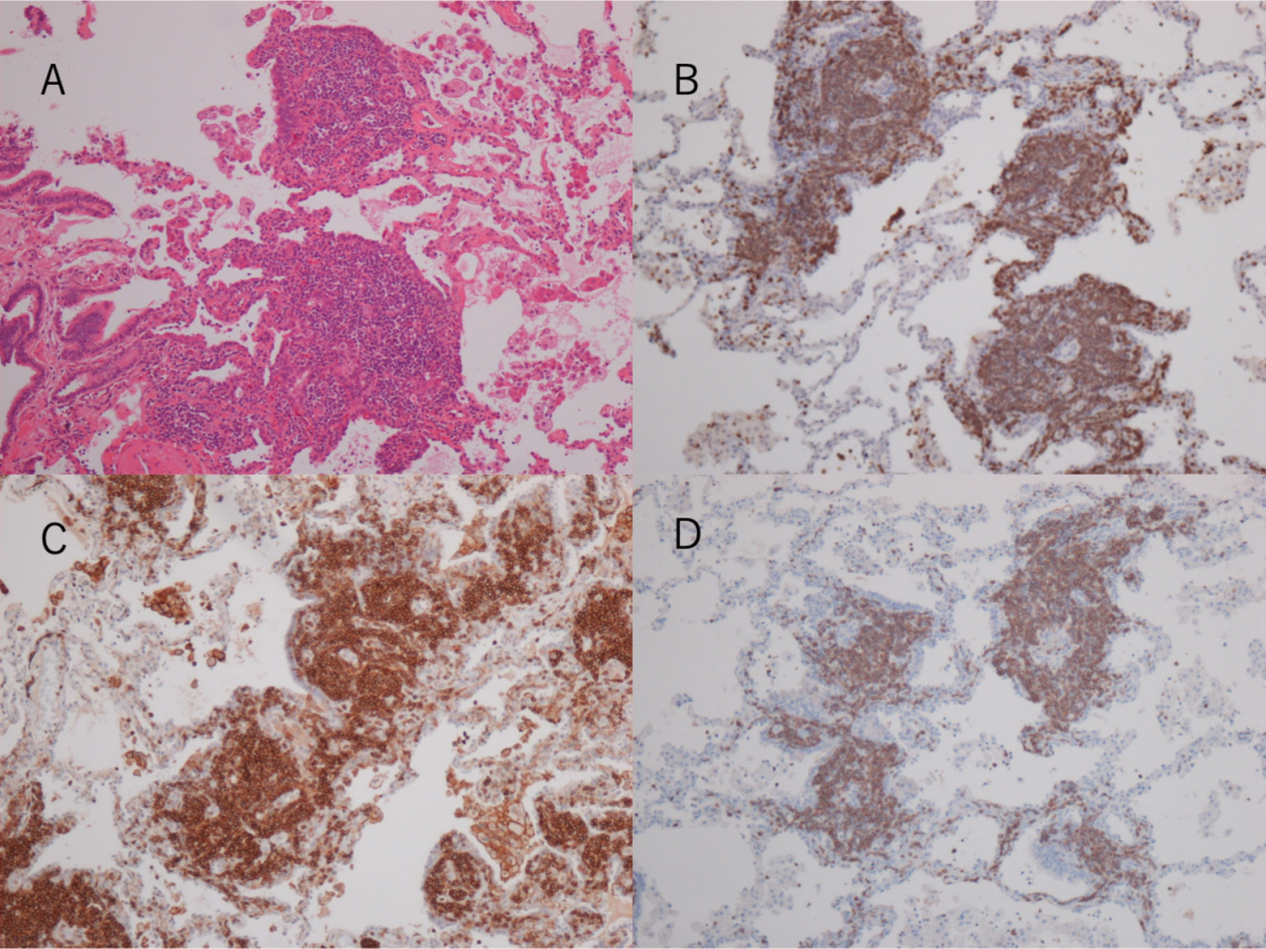
(A) Haematoxylin‐and‐eosin staining of the left lung showing proliferation of atypical lymphocytes and mucosal infiltration. These cells are distributed in the lymphatic channels of the lung, such as the perivascular connective tissue, the respiratory bronchioles, and the connective tissue around the pulmonary arteries. Immunostaining revealed: (B) CD3‐positive T cells; (C) numerous CD4‐positive T cells; and (D) CD5‐positive T cells.

**FIGURE 4:**
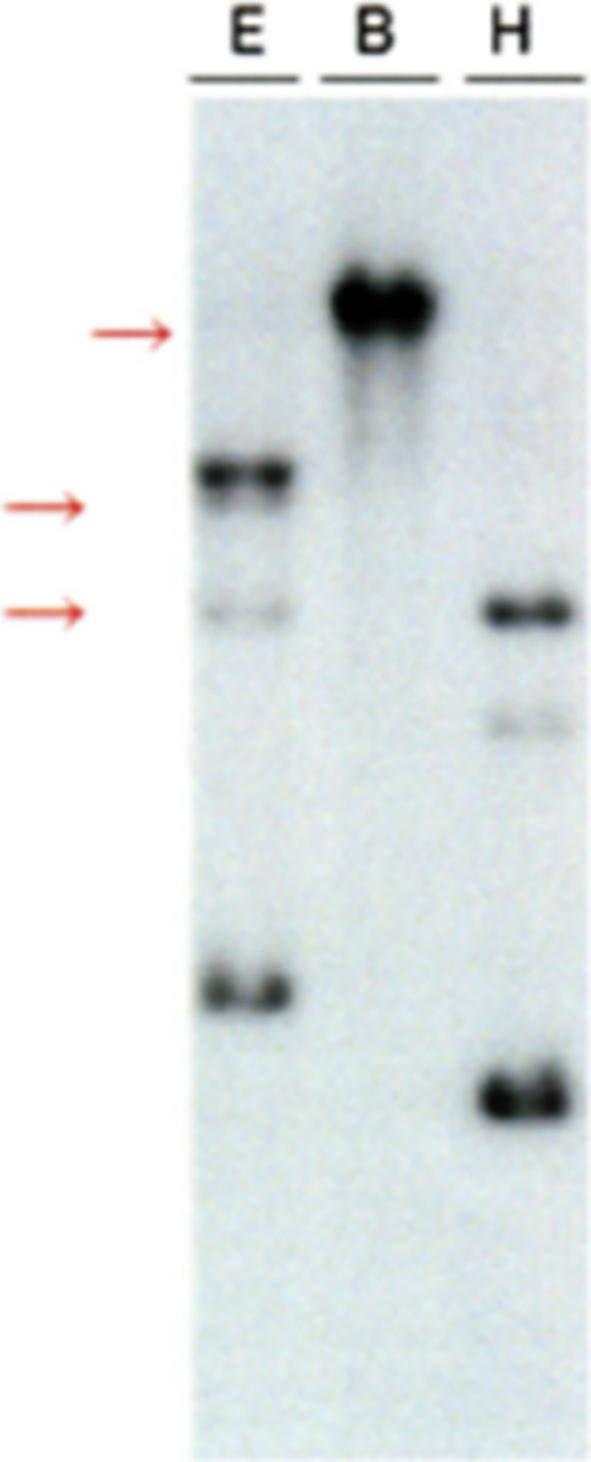
T‐cell receptor β‐chain Cβ1 rearrangement was detected in the thoracoscopic lung biopsy. The red arrows indicate abnormal bands of the T‐cell receptor β‐chain Cβ1 region, suggesting the presence of a monoclonal T‐cell population.

T‐PLL typically presents with hepatosplenomegaly, lymphadenopathy and pleural effusion [2]. Nevertheless, in this case, the disease uniquely manifested as isolated pulmonary lesions. This is the first case with pathological confirmation of isolated lung involvement without other typical manifestations.

## Author Contributions

Yu Kobayashi: patient evaluation, manuscript drafting, image selection, final approval of the manuscript. All authors revised the manuscript and approved the final version of the manuscript.

## Ethics Statement

The authors declare that written informed consent was obtained for the publication of this manuscript and accompanying images and attest that the form used to obtain consent from the patient(s) complies with the Journal requirements as outlined in the author guidelines.

## Conflicts of Interest

The authors declare no conflicts of interest.

## Data Availability

The data that support the findings of this study are available on request from the corresponding author. The data are not publicly available due to privacy or ethical restrictions.
